# New PRSS1 and common CFTR mutations in a child with acute recurrent pancreatitis, could be considered an "Hereditary" form of pancreatitis ?

**DOI:** 10.1186/1471-230X-10-119

**Published:** 2010-10-15

**Authors:** Vito D Corleto, Stefano Gambardella, Francesca Gullotta, Maria R D'Apice, Matteo Piciucchi, Elena Galli, Vincenzina Lucidi, Giuseppe Novelli, Gianfranco Delle Fave

**Affiliations:** 1 Dept. of Digestive and Liver Disease, II School of Medicine, University "La Sapienza", Rome, Italy; 2 Dept. of Biopathology and Diagnostic Imaging, School of Medicine, University of Tor Vergata, Rome, Italy; 3 Research Centre S. Pietro, Ospedale S. Pietro, Rome, Italy; 4 Dept. of Pediatrics, Ospedale Bambino Gesù, Rome, Italy; 5 Fondazione Livio Patrizi, Rome, Italy

## Abstract

**Background:**

acute recurrent pancreatitis is a complex multigenic disease, the diagnosis is even more difficult when this disease develops in a child.

**Case Presentation:**

a 6-years old boy, hospitalized with epigastric pain radiating to the back showed high serum levels of serum amylase, lipase, CRP and erythrosedimentation rate. Several similar milder episodes of pain, followed by quick recovery and complete disappearance of symptoms were reported during the previous 13 months. The child was medically treated and after 7 days with normal clinic and laboratory tests was discharged with a hypolipidic diet. All the known aetiologic hypotheses were excluded by anamnestic investigation, clinical observation and biochemical evaluation, whereas, anatomic abnormality were excluded by a secretin stimulated magnetic resonance (MRI). At the last follow-up visit, (11 months later), the child showed a normal body weight and anthropometric profile, without further abdominal pain. Mutation screening for coding regions of *PRSS1, SPINK1, CFTR *and the new hereditary pancreatitis-associated chymotrypsin C (*CTRC*) genes showed a novel variation, c.541A > G (p.S181G), in the exon 4 of PRSS1 gene and the classical CF p.F508del mutation in the *CFTR. *Both mutations were present in his clinically normal mother and absent in the patient's father.

**Conclusions:**

this report extend the spectrum of PRSS1 mutations, however, the absence of family history of pancreatitis leaves the present case without the hallmark of the hereditary origin of pancreatitis. At the present knowledge it can be only stated that the combined genotype CFTR (F508del)/PRSS1 (S181G) is associated to a mild phenotype of acute recurrent pancreatitis in this child without any further conclusion on its pathogenetic role or prediction on the course of the disease.

## Background

Hereditary Pancreatitis (HP) (OMIM #167800) is a rare autosomal dominant disorder with about 80% of penetrance. The clinical features of HP patients consist in attacks of acute pancreatitis, that in ~80% of individuals starts before 20 years of age (median age 10 years). Progression to chronic pancreatitis also occurs in about ~50% of patients and pancreatic cancer may develop by 15 years of age in about ~40% of affected individuals [1,2]. In 1996, a correlation was first reported between the development of the disorder and hereditary factors with the *PRSS1 *gene, that encodes for the human cationic trypsinogen [3,4]. Mutations and copy number variation (CNV) in the *PRSS1 *gene are responsible for the increase of autocatalytic conversion of trypsinogen to active trypsin that leads to autodigestion of the organ [5,6].

Chronic pancreatitis has also been associated with mutations in other genes such as *SPINK 1 *(serine protease inhibitor Kazal Type I) [7], *CFTR (*Cystic Fibrosis transmembrane conductance regulator) [8], *CTRC *(chymotrypsin C) [9]. Contemporary mutations, in these genes, trigger a distinctly greater risk of developing chronic pancreatitis [10-12]. While the polymorphism p.G191R in *PRSS2 *(cationic trypsinogen type 2) gene protects against chronic pancreatitis [13].

The present report focuses on the finding of a new heterozygous mutation in the *PRSS1 *gene, associated with p.F508del mutation in *CFTR*, in a child presenting acute recurrent pancreatitis.

## Case Presentation

The case is described of a 6-year-old boy, examined in the Emergency Department on account of acute abdominal pain, intense nausea and mild fever (37.5°C). The clinical history revealed 6 similar episodes during the previous 13 months, some managed at home and other with brief hospitalization. During the very first episode in which he also presented a white blood count of 22,000 (88% neutrophils), appendicectomy was performed. A review of previous medical charts revealed that amylase had been evaluated only twice in the past and, on both occasions, it was slightly increased (twice the normal value). However, no further investigation had been performed due to rapid recovery and complete disappearance of abdominal pain. When coming to our attention, the boy appeared to be suffering, with knees drawn up to his chest, and with epigastric pain radiating to his back, of post-prandial onset. Furthermore, the child presented moderate abdominal distension, however, all vital signs were within normal limits. Laboratory studies were all within the normal range, with the exception of serum amylase which was 1373 U/L (n.v. 0-95 U/L), lipase 1050 U/L (n.v. 13-60 U/L), C-reactive protein (CRP) 95 mg/L (n.v. 0-10 mg/L), erythrosedimentation rate (ESR) 74 mm/h (n.v. 0-20 mm/h). The child was treated with intravenous (i.v.) fluids plus 20 mg of proton pump inhibitor (PPI) i.v. An abdominal sonogram showed increased pancreatic volume with diffuse oedema, gallbladder was normal. The following day, laboratory tests showed overall decreased values: amylase 650 U/L, lipase 350 U/L, CRP 30 mg/L. Fluids i.v. were administered for 4 days after which amylase, lipase, CRP and ESR returned close to normal values and a liquid light meal was administered with good results. After 7 days of hospitalization, with normal laboratory tests and without abdominal pain, the child was discharged with a hypolipidic diet, 20 mg PPI daily and pancreatic enzyme supplementation (10,000 U × 6/day) per os. both for two more weeks. Taking into consideration all the aetiologic hypotheses, blunt trauma, metabolic, infectious, drug and systemic causes were excluded, by means of accurate anamnestic investigations and clinical observations and by further laboratory evaluations (calcium, glucose and triglycerides were always normal; Mumps, Cytomegalovirus, Coxsackie B, Herpes Simple virus antibodies were negative). In the attempt to exclude any anatomic abnormality, secretin-stimulated magnetic resonance imaging (MRI) of the pancreas was programmed as an outpatient. At follow-up visits, the child presented stable body weight, good appearance, with no further episodes of abdominal pain, continuing a strictly controlled of hypolipid diet. The secretin MRI, performed 2 months later, showed an overall increased thickness of the parenchyma of the entire pancreas with aspecific irregular signal intensity in the head region. Following secretin stimulation, the main pancreatic duct appeared to be normal, with a normal papilla in the second part of the duodenum that was normally filled, 10 minutes after the secretin infusion. At the last follow-up visit, ~11 months after the last acute pancreatic attach, the child presented a normal anthropometric profile with normal body weight, and no further episodes of abdominal pain were reported. However, two new short episodes of abdominal pain have been recently reported with a slight increase of only lipase levels (110 U/L; n.v. 13-60) following the last one.

**Figure 1:**
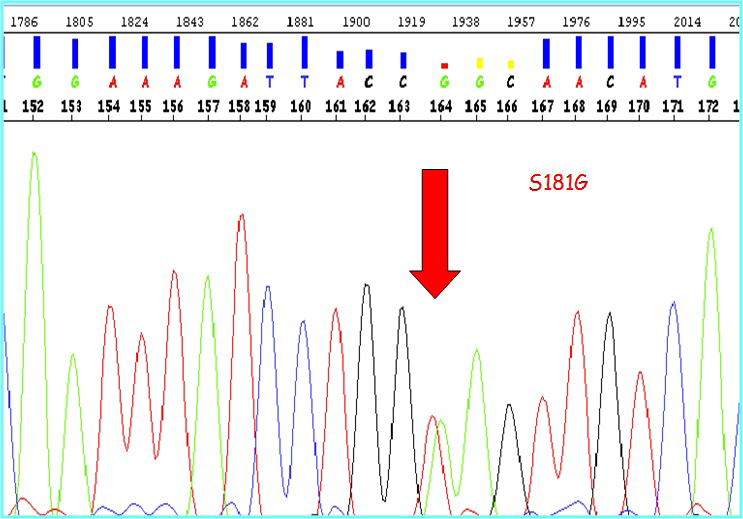
Sequence chromatogram of the PRSS1 mutation p.S181G.

Given the unexplained episode of pancreatitis in a child, it was decided to investigate point mutations in the cationic trypsinogen gene (*PRSS1*) underlying HP, although no data were available suggesting familial pancreatitis in this patient. *CFTR*, *SPINK-1 *and the new HP-associated chymotrypsin C (*CTRC*) gene were also analysed. While genetic testing revealed normal *SPINK1 *and *CTRC *genes, a novel heterozygous variation, c.541A > G (p.S181G), in exon 4 of *PRSS1 *gene was revealed (Figure 1). This transition was never been detected in 100 unrelated healthy controls, suggesting that this variation is a putative mutation (http://www.ncbi.nlm.nih.gov/sutils/blink.cgi?mode=result&pid). The patient also showed the classic p.F508del mutation, in a heterozygous state, in the *CFTR *gene. However, no other nucleotide variations or genomic rearrangements were detected in this gene following careful examination. Both mutations were absent in the patient's father but present in his clinically healthy mother.

To explain the discordance in phenotype, between mother and proband, the following possibilities were taken into consideration: i) presence, in the proband or the father's genome, of gross deletions/insertions in the pancreatitis causative genes; ii) presence of active modifier factors such as protective polymorphism p.G191R in the PRSS2 gene; iii) non paternity, and iv) environmental effects.

To exclude the first hypothesis, we applied aCGH (array-based Comparative Genomic Hybridization) to detect macro deletions and macro insertions on the entire genome of the father's and proband's DNA. No significant alterations were detected (data not shown). The second and third hypotheses were ruled out by complete sequence analysis of the *PRSS2 *gene and testing of paternal micro-satellites (data not shown). Furthermore, a detailed dietary history of the child showed high frequency of consumption of fat food prior to the onset of the acute pancreatitis episodes.

## Methods

The parents were constantly informed regarding the interpretation of the clinical and genetic investigations and always gave their written consent both to the procedures and the laboratory investigations. *PRSS1*, *PRSS2*, *SPINK1*, *CTRC *and *CFTR *coding regions amplification and sequencing were performed on an ABI 3130 Genetic analyzer, according to previously reported conditions [7,9,13-15], (Applied Biosystems; Foster City, CA, USA).

Analysis of *CFTR *rearrangements was performed using a quantitative PCR followed by capillary electrophoresis (Multiplex ligation-dependent probe amplification, MLPA SALSA Kit, MRC-Holland, The Netherlands). aCGH was performed by Agilent's Oligonucleotide Array-Based CGH for Genomic DNA 4 × 44 K.

## Discussion and Conclusions

The present case represents a rare condition in a child with recurrent acute pancreatitis allegedly determined or favoured by a compound heterozygous mutations, p.F508del in *CFTR *and a novel maternal transmitted mutation, p.S181G, in exon 4 of the *PRSS1*. This report potentially extends the spectrum of the *PRSS1 *mutation. In fact, all pancreatitis-associated *PRSS1 *mutations discovered appear to cluster in the N-terminal half of the molecule encoded by exons 2 and 3. Whereas, the present p.S181G is the first mutation detected in exon 4 corresponding to an amino acid position inside two disulphide bridges, amino acids: 139-206 and 171-185. Although it is well known that chronic pancreatitis is predisposed by heterozygosity for *CFTR *mutations, little is known about the combination of *CFTR *mutations with *PRSS1 *or *SPINK1 *mutations. To date, only a few cases have demonstrated that pancreatitis is caused by simultaneous *CFTR *and *SPINK1 *mutations (5.5%), of *PRSS1 *and *SPINK1 *(1.3%), and of *PRSS1 *and *CFTR *(1.8%). No information concerning clinical features of these patients are available. Between 60% and 80% of HP patients carry a pathogenic PRSS1 mutation. Lower penetrance and perhaps, in some cases, modifier genes have been identified, particularly the CFTR and SPINK1 [16].

In the present report, the combined genotype F508del/S181G is associated with acute pancreatitis episodes in the 6-year-old proband, but not in his 37-year-old mother, who carries the same genotype, but without any pancreatic symptoms, biochemical or ultrasound pancreatic alterations.

Following the experimental exclusion of gross deletions/insertions in the pancreatitis causative genes in the father, of a case of non paternity, and of the presence of other mutations in pancreatitis relevant genes, it is tempting to suggest that the phenotypic discordance may be due to unknown combination of genetic and/or environmental factors.

Therefore, it could be a potential late onset of pancreatic disease in the proband's mother, or a case of incomplete penetrance of a genotype composed by two mutations (up to 20% of *PRSS1 *gene carriers may remain symptom-free) [14]. In addition, it cannot be excluded that this genotype-phenotype discordance is due to unidentified modifier genes, relevant for the development of pancreatitis. On the other hand, the finding that a hypolipidic diet has been able to prevent any further pancreatic attack, suggests that diet could have a great importance between the proband and his mother phenotype. Similar cases of phenotypic discordance represent an unresolved genetic aspect that will be addressed in the future i.e. why some carriers of PRSS1 mutation remain completely healthy whereas their relatives with the same mutation develop various grade of disease [1]. However, the absence of family history of pancreatitis leaves the present case without the hallmark of the hereditary origin of pancreatitis in the present child. In conclusion, functional studies are mandatory to understand if and/or how *PRSS1 *mutation S181G could be involved in determining recurrent pancreatitis or if it needs to be combined with other HP causing genes such as CFTR mutations. At the moment, the combined genotype F508del/S181G could be only associated to a mild phenotype of acute recurrent pancreatitis as happened in the present child. Moreover, on the base of the present report we cannot establish whether PRSS1 S181G gene mutation has a pathogenetic role on the child pancreatic disease nor any prediction on the course of the disease.

## Authors' contributions

VDC and SG design and drafted the manuscript and carried out the molecular genetic studies; FG, MRD participated in the molecular genetic studies and sequence alignment; MP, EG and VL followed the clinical part of the study; GN and GDF conceived of the study and its coordination.

All authors read and approved the final manuscript.

## Pre-publication history

The pre-publication history for this paper can be accessed here:

http://www.biomedcentral.com/1471-230X/10/119/prepub
